# Triadic shared decision making in emergency psychiatry: an explorative study

**DOI:** 10.1186/s12888-025-06640-7

**Published:** 2025-03-05

**Authors:** G. C.Roselie van Asperen, R. F.P. de Winter, C. L. Mulder

**Affiliations:** 1 Parnassia Psychiatric Institute, Dynamostraat 18, Rotterdam, 3083 AK the Netherlands; 2 Epidemiological and Social Psychiatric Research institute, Department of Psychiatry, Erasmus University Medical Center, Rotterdam, PO Box 2040, 3000 CA the Netherlands; 3 Mental Health Institute Rivierduinen, Leiden, PO Box 405, 2300 AK the Netherlands; 4 Department of Psychiatry & Neuropsychology, Maastricht University, Maastricht, P.O. Box 616, 6200 MD the Netherlands; 5 Psychology and Pedagogy, Vrije Universiteit Amsterdam, Amsterdam, P.O. Box 7057, 1007 MB the Netherlands; 6 Erasmus Medical Center, Dr. Molewaterplein 40, Rotterdam, 3000 CA the Netherlands

**Keywords:** Triadic shared decision making, Emergency psychiatry, Acute mental health care, Inpatient care, Outpatient care

## Abstract

**Background:**

This study aims to understand the complex triadic shared decision-making process in psychiatric emergency services, focusing on the choice between inpatient and outpatient care post-triage. It also identify scenarios where patient or significant others’ preferences override clinical judgment.

**Methods:**

Conducted in the greater Rotterdam area, Netherlands, this explorative study surveyed patient and significant others’ preferences for voluntary or involuntary admission versus outpatient treatment, alongside professionals’ clinical indications. Descriptive statistics were used to profile participants, and preference data were used to categorize groups, revealing patterns of agreement.

**Results:**

Among 5680 assessments involving significant others, four groups emerged: agreement among the triad on in- or outpatient care (48.2%), patient disagrees (38.5%), significant others disagree (11.0%), and professionals disagree (2.3%). Professionals’ recommendations were followed more frequently (57.0%) than patient (9.4%) or significant others’ preferences (11.0%).

**Conclusions:**

We observed that consensus could often be reached among the members of the triad on inpatient or outpatient care following triage. Disagreements typically occurred when patients preferred outpatient care while others favoured inpatient care, or when significant others advocated for inpatient care while others preferred outpatient care. While professionals’ recommendations held the most influence, they could be overridden in cases where valid criteria mandated involuntary care.

## Background

Shared decision-making (SDM) is a collaborative approach wherein healthcare professionals and patients engage in consensus-building to ascertain the treatment course and reach a treatment agreement [1]. The aim of this approach, which regards the participation of individuals experiencing mental health conditions as an ethical imperative [2], is to improve treatment outcomes, for which there is evidence [3, 4]. Involvement of family, friends, neighbours or other carers - i.e. ‘significant others’ – in triadic shared decision making, serves to establish SDM by providing supplementary information and comfort [5]. Participation of significant others is defined as the acknowledgment of their contributions and the incorporation of their background information into the decision-making process [6]. Their participation has been associated with reduced inpatient admissions, shorter inpatient stays, and improved quality-of-life outcomes reported by patients [7].

The transition from institution-based treatment to community-based care introduces shifts in the roles of significant others [8]. Significant others take on responsibility in domains that may be inadequately addressed by healthcare professionals [9], potentially playing a pivotal role in managing various aspects of daily life, such as finances, housing, and social interactions [10, 11]. As patients receive care within their domestic environments, significant others often assume responsibility for the provision of support. However, these individuals frequently have feelings of blame for having caused mental health problems and are at higher risk of experiencing health-related, emotional, and financial burdens themselves [6, 12]. Consequently, the implementation of significant others’ involvement in the care process can be challenging [13, 14].

The deployment of emergency psychiatry constitutes a particularly challenging setting for the participation of significant others in the SDM process. Risk reduction and crisis management are often prioritized, frequently resulting in inpatient care. The focus on safety and the possibility of inpatient care can complicate the engagement of significant others [14]. One study found the will of significant others for inpatient care as the most important determinant in the decision between in- and outpatient care [15]. In an observational study of a Crisis Resolution and Home Treatment Team (CRHT) in the Netherlands, the involvement of significant others was observed in two-thirds of cases, facilitated through the use of a structured motivational model [16]. Notably, it was observed that treatment outcomes were similar, regardless of the in- or exclusion of significant others, even in instances where patients seem to be reluctant at first. A qualitative study described the needs of patients and significant others during psychiatric emergency services [17]. Effective communication with both patients and significant others was found to be important to enhance cooperation. Healthcare professionals must be able to tailor their approach. A conceptual review described the challenges professionals are confronted with in the course of involving significant others in psychiatric emergency services [18]. The complexity of this involvement arises from the diverse expectations and needs of patients, significant others, and mental healthcare professionals, coupled with the delicate decision-making process regarding the choice between in- and outpatient care.

The primary aim of this study is to disentangle the SDM process, primarily exploring the relative impact of the preferences of significant others, and the additional impact of patients and professionals, on the decision for in- or outpatient care after triage, taking the severity of the patients’ condition into account. As a secondary aim, this study describes the specific scenarios in which either patient or significant others’ preferences supersede the professionals’ clinical judgment.

## Methods

### Study design

This was a retrospective and explorative study using data from the electronic health record (EHR) of the emergency psychiatric service.

### Setting and participants

**Fig. 1:**
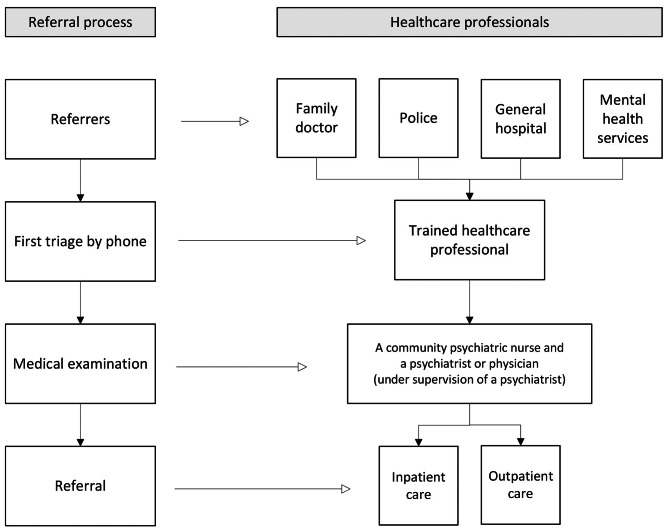
Referral process of the emergency psychiatric services in Rotterdam

This study was conducted within the greater Rotterdam area, situated in the southwestern region of the Netherlands. In this area, the emergency psychiatric service is responsible for the triage when confronted with individuals experiencing a mental health crisis. The primary sources of referrals to these services are family doctors, the police, general hospitals, and mental health services [19]. A schematic representation of the referral process is provided in Fig. 1. The emergency psychiatric service is tasked with responding rapidly (at least within 24 h) to sudden changes in patients’ mental well-being or instances of behavioural loss of control, which may include suicidal crises. The initial triage consists of telephone consultation and is performed by a trained mental healthcare professional to ascertain the necessity for a comprehensive face-to-face evaluation and crisis intervention. When after the telephone consultation it is decided that such a comprehensive evaluation and intervention is needed, a community psychiatric nurse and either a psychiatrist or a physician, the latter working under the supervision of a psychiatrist, goes to visit the patient. This medical examination primarily occurs at the patient’s home but may also take place at a mental health facility or a (psychiatric) hospital. The psychiatrist assumes responsibility for rendering a psychiatric diagnosis. The involvement of significant others is encouraged during this evaluation, and their perspectives and preferences are factored into the decision-making process. It is during this SDM process that the determination regarding in- or outpatient care is indicated.

The study’s participants comprised individuals aged 18 years and older who had undergone assessment by the emergency psychiatric service during the period spanning January 2015 to December 2019. This approach allowed for participants to be included multiple times in the series. A significant other had to be present during the assessment for the case to enter the study.

### Data collection

Under the Netherlands Agreement on Medical Treatment Act (WGBO), patient record research does not require informed consent if individual patients cannot be identified from the data. Our study received approval from the internal scientific research committee of Parnassia Groep, which determined that the study was outside the scope of the Netherlands Medical Research Involving Human Subjects Act (WMO) and confirmed that informed consent was not necessary. In compliance with applicable Dutch laws, all researchers were bound by strict confidentiality agreements [20].Data were retrospectively gathered from the WebRAAP (Web-based Registration and Advisory system for Acute Psychiatry) EHR, designed for the documentation of the activities of the emergency psychiatric service [21]. Prior to access by the authors, these data underwent complete anonymization, conducted by Myosotis ICT, a trusted data processing firm. The collected data encompassed various characteristics: patient characteristics such as age, gender, living situation and primary diagnosis, and the patient’s as well as the significant others’ preference for either (in)voluntary admission to a psychiatric hospital, or outpatient treatment such as Intensive Home Treatment or other outpatient care services, and the professionals’ clinical indication for these services. The preferences of the triad members were recorded during the medical examination by a healthcare professional conducting the assessment. If the patient or significant others did not express a preference, this was recorded as missing data. However, this procedure could introduce bias. Misunderstandings arising from cultural differences, language barriers, or differing communication styles may have resulted in inaccurate documentation. To minimize this potential bias, the documentation was cross-checked between the two healthcare professionals involved in the assessment.

In addition to this, the psychiatric nurse and medical doctor filled out the Severity of Psychiatric Illness (SPI) scale, which was administrated in Dutch [15]. The SPI is an observer rated scale, comprising of 14 dimensions, evaluating the severity of psychiatric illness, and employs a 4-point scale ranging from 0 (indicating no problems) to 3 (indicating severe problems) [22, 23]. The 14 dimensions assessed by the SPI scale encompass suicide potential, danger to others, severity of psychiatric symptoms, problems with self-care, substance abuse, medical complications, social complications, problems with professional functioning, problems with living conditions, problems with motivation for treatment, problems with compliance, problems with disease awareness, problems with family involvement, and persistence of problems. Due to the asymmetric distribution of the data, these variables were dichotomized into “no problem” and “small to severe problem.”

Note that in this study, the indicated level of care following the triage process was used for determining (dis)agreement within the SDM process, rather than the level of care that could actually be provided. The determination of the indicated level of care is determined mainly by clinical factors, because e.g. bed availability is not taken into account in this SDM process. Conversely, however, the level of care that could actually be provided was also determined by contextual factors such as bed availability. This study will present the percentage of (dis)agreements within the triad on the indicated level of care to eliminate as much as possible the impact of contextual factors. Additionally, the realization of care—the level of care actually provided—is described to explore the impact of the triad’s preferences on the final outcomes.

### Data analysis

Statistical analyses were performed using SPSS (Statistical Package for the Social Sciences) version 26.0 (SPSS Inc., Chicago, IL). Initially, descriptive statistics were computed to provide a profile of the study’s participants. This analysis involved the examination of demographic variables, such as gender and age. Furthermore, the preferences for either in- or outpatient care stated by the parts of the triads were used to stratify groups, allowing for the exploration of distinct patterns of agreement among these groups. The SPI scale was used to assess the severity of psychiatric illness across 14 dimensions for the diverse subgroups.

The SPI scale was used to describe its influence on care decisions, particularly in situations where patient or significant others’ preferences superseded the professionals’ preference based on clinical judgment. This analysis described the relationship between the SPI dimensions and the choice for in- or outpatient care. It sought to clarify the composition of cases and the severity of psychiatric symptoms to impact the selection of care options. To determine a difference among groups, exploration was conceptualized as different tests of the same hypothesis [24], and p-values were intentionally omitted in accordance with established methodology [25].

## Results

Over a period of 4 years (2016–2019), the emergency psychiatric service undertook 12,470 assessments of patients aged 18 and older. Significant others were present during almost half of these assessments (45.5%), leading to 5680 assessments meeting the inclusion criteria for this study. Table 1 shows the patient characteristics of the patients assessed by the psychiatric emergency services, divided into both groups. The groups exhibited comparability across most factors, with the primary distinguishing factor being the living situation. Notably, the presence of significant others was more prevalent among patients living with family in contrast to other living situations.

**Table 1:** Characteristics of patients assessed by the psychiatric emergency services

Factors	Significant others present	Significant others not present	Total
Total n*	5680 (45.5)	6790 (54.5)	12470 (100)
Age: mean (SD)	45.20 (20.02)	41.85 (16.32)	43.38 (18.17)
Gender: *n*, female	2815 (49.6)	2916 (42.9)	5731 (46.0)
Native language Dutch: *n*	3133 (55.2)	3521 (51.9)	6654 (53.4)
Living situation			
Alone: *n*	1556 (27.4)	2616 (38.5)	4172 (33.5)
With family: *n*	2776 (48.9)	1588 (23.4)	4364 (35.0)
Institution: *n*	133 (2.3)	443 (6.5)	576 (4.6)
Other/unknown: *n*	1096 (19.3)	1658 (24.4)	2754 (26.9)
Without residence: *n*	119 (2.1)	485 (7.2)	604 (3.7)
Primary diagnosis			
Depressive disorder: *n*	981 (17.3)	1036 (15.3)	2017 (16.2)
Bipolar disorder: *n*	446 (7.9)	351 (5.2)	797 (6.4)
Anxiety disorder: *n*	206 (3.6)	203 (3.0)	409 (3.3)
Post traumatic stress syndrome: *n*	104 (1.8)	208 (3.1)	312 (2.5)
Psychosocial problems: *n*	105 (1.8)	228 (3.6)	333 (2.7)
Adjustment disorder: *n*	151 (2.7)	291 (4.3)	442 (3.5)
Personality disorder: *n*	379 (6.7)	733 (10.8)	1112 (8.9)
Psychotic disorder: *n*	1820 (32.0)	2044 (30.1)	3864 (31.0)
Organic disorder: *n*	568 (10.0)	260 (3.8)	828 (6.6)
Alcohol-related disorder: *n*	253 (4.5)	453 (6.5)	696 (5.6)
Other substance-related disorder: *n*	176 (3.1)	320 (4.7)	496 (4.0)
Other: *n*	443 (7.8)	559 (8.2)	1002 (8.0)
None/diagnoses deferred: *n*	48 (0.8)	114 (1.7)	162 (1.3)

*%, percentage of the total group

**Fig. 2:**
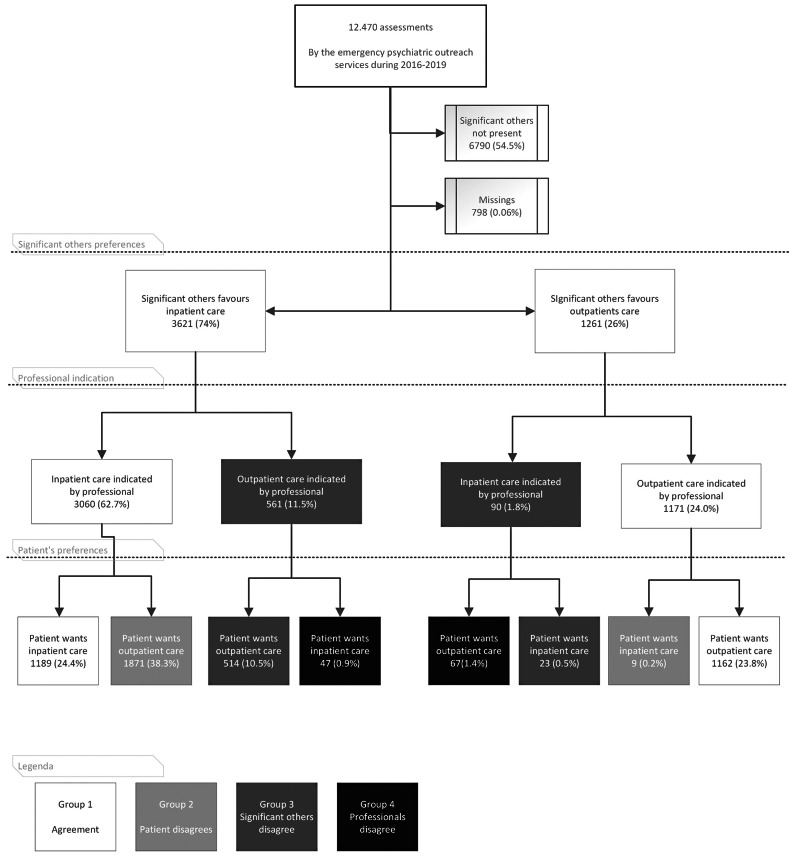
The distribution of the 4 groups on (dis)agreement in the triad

Figure 2 shows the distribution of the preferences of the patients and the indication of the professionals, divided by the wish of the significant others, whether they preferred in- or outpatient care. The figure consists of white boxes representing agreement and grey boxes representing disagreement in the triad. This distribution leads to 4 groups: agreement on in- or outpatient care, patient disagrees, significant others disagree or professionals disagree. Consensus is achieved in most cases when outpatient care is selected as the preferred level of care within the triad. Disagreements tend to emerge when a patient wants outpatient care while the remaining triad wants inpatient care (38.3%) or when significant others want inpatient care while the remaining triad wants outpatient care (10.5%).

For additional details on the four groups, please refer to Table 2. This table shows the distribution of the 4 groups on (dis)agreement with in- or outpatient care in the triad and the realization of in- or outpatient care.

**Table 2:** Distribution of (dis)agreement on the indication and realization of in- and outpatient care

Group	Total of 4882	(Dis)agreement with in- or outpatient care	Realization of in- or outpatient care
**Agreement on in- or outpatient care**: n*	2351 (48.2)	Inpatient care:	Outpatient care: 72 (6.1)
		1189 (50.6)	Inpatient care: 1117 (93.9)
			*Part involuntary: 174 (15.6)*
		Outpatient care:	Outpatient care: 1160 (99.8)
		1162 (49.4)	Inpatient care: 2 (0.2)
			*Part involuntary: 1 (50.0)*
**Patient disagrees with the other two components of the triad**: ***n***	1880 (38.5)	Only the patient wants inpatient care:	Outpatient care: 9 (100)
		9 (0.5)	Inpatient care: 0
		Only the patient wants outpatient care:	Outpatient care: 177 (9.5)
		1871 (99.5)	Inpatient care: 1694 (90.5)
			*Part involuntary: 1480 (87.4)*
**Significant others disagree with the other two components of the triad**: ***n***	537 (11.0)	Only the significant others want inpatient care:	Outpatient care: 465 (90.5)
		514 (95.7)	Inpatient care: 49 (9.5)
			*Part involuntary: 35 (71.4)*
		Only the significant others want outpatient care:	Outpatient care: 6 (26.1)
		23 (4.3)	Inpatient care: 17 (73.9)
			*Part involuntary: 3 (17.6)*
**Professionals disagree with the other two components of the triad**: ***n***	114 (2.3)	Only the professionals indicate inpatient care:	Outpatient care: 34 (51.7)
		67 (58.8)	Inpatient care: 33 (49.3)
			*Part involuntary: 25 (75.8)*
		Only the professionals indicate outpatient care:	Outpatient care: 32 (68.1)
		47 (41,2)	Inpatient care: 15 (31.9)
			*Part involuntary: 2 (13.3)*

*%, percentage of the total group of patients

### Agreement on in- or outpatient care

Agreement among the triad regarding in- or outpatient care is shown in the first and the last columns of Fig. 2 and was achieved in 2351 assessments, representing 48.2% of cases. When all three involved parties reached an agreement, the selected care level was nearly always implemented as decided.

### Patient disagrees

The patient held a different viewpoint from both the significant others and the professionals and stands alone advocating for either in- or outpatient care. The disagreement is shown in the second and second-to-last columns of Fig. 2, totalling 1880 assessments, amounting to 38.5% of cases. Predominantly, the patient favoured outpatient care (99.5%), while the other two components of the triad favoured inpatient care. In most of these cases (90.5%), inpatient care was realized despite the patient’s preference for outpatient care. Disagreement on outpatient care in this group occurred scarcely (0.5%) and always resulted in outpatient care.

In the group where the patient wanted outpatient care and the other two components of the triad wanted inpatient care, we saw the highest proportion of involuntary admissions (87.4%).

Despite the disagreement, the patient’s preference was granted in 9.4% of the assessments. This only occurred when the patient favoured outpatient care and the other two components of the triad favoured inpatient care.

Exploring the particular subset where the patient’s preference supersedes both the significant others’ viewpoint and the professionals’ clinical assessment, the case mix characteristics quantified using the SPI showed few differences. For additional details on this group, see Table 3. Within the group where the preference of the patient was granted, patients scored less on suicide potential (13.0% vs. 19.9%) and less on danger to others (17.5% vs. 34.2%) compared to the group where the preference of the patient was not granted.

**Table 3:** SPI of patients who disagree with the other two components of the triad

Severity of psychiatric illness	Preference of the patient was granted (outpatient)	Preference of the patient was not granted (inpatient)
Total N*	177 (9.4)	1703 (91.6)
**Suicide potential**: *n*	23 (13.0)	339 (19.9)
**Danger to others**: *n*	31 (17.5)	582 (34.2)
**Severity of psychiatric symptoms**: *n*	69 (39.0)	519 (30.5)
**Problems with self-care**: *n*	41 (23.2)	478 (28.1)
**Substance abuse**: *n*	31 (17.5)	233 (13.7)
**Medical complications**: *n*	19 (10.7)	247 (14.5)
**Social complications**: *n*	50 (28.2)	449 (26.4)
**Problems with professional functioning**: *n*	48 (27.1)	455 (26.7)
**Problems with living conditions**: *n*	27 (15.3)	218 (12.8)
**Problems with motivation for treatment**: *n*	36 (20.3)	346 (20.3)
**Problems with compliance**: *n*	31 (17.5)	267 (15.7)
**Problems with disease awareness**: *n*	114 (64.4)	1187 (69.7)
**Problems with family involvement**: *n*	6 (3.4)	27 (1.6)
**Persistence of problems**: *n*	44 (24.9)	324 (19)

*%, percentage of the total group of patients

### Significant others disagree

In 537 assessments, amounting to 11.0% of cases, the significant others held a different viewpoint from both the patient and the professionals and stand alone advocating for either in- or outpatient care. The disagreement is shown in the third and sixth columns of Fig. 2. Predominantly, the significant others favoured inpatient care (95.7%), while the other two components of the triad favoured outpatient care. In most of these cases (90.5%), outpatient care was realized despite the significant others’ preference. Disagreement on outpatient care in this group occurred rarely (4.3%).

Despite the disagreement, the significant others’ preference for inpatient care was granted in 9.5% of the assessments, the preference for outpatient care was granted in 26.1% of the assessments. This equates to a 11.0% allowance rate for the significant others’ preference in all assessments where the significant others express disagreement.

Exploring the particular subset where the significant others’ preference supersedes both the patient’s viewpoint and the professionals’ clinical assessment, the case mix characteristics quantified using the SPI showed few differences. For additional details on this group, see Table 4. Within the group where the preference of the significant others was granted, patients scored higher on suicide potential (21.8% vs. 14.3%), higher on danger to others (20.0% vs. 7.7%) and higher on problems with motivation (23.6% vs. 12.7%) compared to the group where the preference of the significant others was not granted.

**Table 4:** SPI of patients from whom significant others disagree with the other two components of the triad

Severity of psychiatric illness	Preference of the significant others was granted	Preference of the significant others was not granted
**Total N**	55 (10.2)	482 (89.8)
**Suicide potential**: *n*	12 (21.8)	69 (14.3)
**Danger to others**: *n*	11 (20.0)	37 (7.7)
**Severity of psychiatric symptoms**: *n*	26 (47.3)	199 (41.3)
**Problems with self-care**: *n*	14 (25.5)	99 (20.5)
**Substance abuse**: *n*	8 (14.5)	64 (13.3)
**Medical complications**: *n*	10 (18.2)	76 (15.8)
**Social complications**: *n*	14 (25.5)	115 (23.9)
**Problems with professional functioning**: *n*	18 (32.7)	115 (23.9)
**Problems with living conditions**: *n*	5 (9.1)	46 (9.5)
**Problems with motivation for treatment**: *n*	13 (23.6)	61 (12.7)
**Problems with compliance**: *n*	12 (21.8)	43 (8.9)
**Problems with disease awareness**: *n*	33 (60.0)	254 (52.7)
**Problems with family involvement**: *n*	3 (5.5)	8 (1.7)
**Persistence of problems**: *n*	18 (32.7)	96 (19.9)

*%, percentage of the total group of patients

### Professionals disagree

Occasionally, the professionals held a different indication from both the patient and the significant others and stand alone advocating for either in- or outpatient care in 114 assessments, amounting to 2.3% of cases, with a nearly equal distribution between the indications for in- and outpatient care. This can be found in the fourth and fifth columns of Fig. 2.

When only the professionals indicated inpatient care, the realization of both in- and outpatient care was almost equally distributed. However, in cases where only the professionals indicated outpatient care, outpatient care was realized in 67.4% of the assessments.

Proportionally, the professionals’ indication was granted more frequently (57.0%) compared to the preference of both the significant others (11.0%) and the patient (9.4%).

## Discussion

Significant others were present in nearly half of all assessments conducted by the emergency psychiatric service (45.5%). Our exploration of the triadic decision-making process during an assessment by the emergency psychiatric service revealed that consensus was reached in almost half of the assessments (48.2%).

Disagreements on the indication of level of care tend to emerge when patients want outpatient care while the remaining part of the triad wants inpatient care (38.3%) or when significant others want inpatient care while the remaining triad wants outpatient care (10.5%). Patients tended to resist inpatient care when significant others requested it. This emphasizes the findings of a conceptual review, illustrating the diverse expectations and requirements among patients, significant others, and healthcare professionals in emergency psychiatric care [18]. The patient, as an individual in acute care, occupies a vulnerable position and faces a risk of identity loss, potentially becoming a passive recipient of interventions. The significant other serves as a resource for both the patient and the healthcare professional. The healthcare professional is tasked with carefully balancing the patient’s preferences alongside the preferences of the significant other.

The patient holds the most vulnerable position within the triad, exhibiting the highest level of disagreement with the rest of the triad. The patient’s preference was granted in 9.4% of the assessment when there was disagreement in the triad. In contrast, the significant others’ preference was granted in 11.0% of cases, while the professionals’ indication was granted in 57.0% of assessments when there was disagreement in the triad. These findings seemingly contradict an earlier study that identified the significant others’ preference for inpatient care as the most important factor influencing the decision between in- and outpatient care [15]. However, in this analysis the category “family or friends do not favour admission” included all cases with low problem severity and all cases where family or friends were not present. So, in many cases the variable “family or friends’ preference for admission” was not a predictor of inpatient care because the significant others were not present or opted for outpatient treatment in full agreement with the health care professionals.

Our study’s outcomes do align with a qualitative study examining the engagement of significant others of individuals diagnosed with serious mental illness (SMI) in SDM [26]. This research revealed that the decision-making process is not democratic. While there is a growing recognition of the necessity to involve significant others, SDM has not been fully achieved or implemented in practice. The professionals hold the strongest position within the triad, which logically derives from their role in addressing crisis situations and possessing the expertise to navigate such circumstances. However, a review focusing on the involvement of significant others uncovered that they often perceived mental health professionals to have negative attitudes regarding their involvement [27]. Consequently, it becomes important for professionals to recognize the significance of involving significant others and to handle this involvement with sensitivity, acknowledging the importance of the involvement in the overall care process.

In certain scenarios, the professionals allow either the patient’s or the significant others’ preferences to supersede their clinical judgment. This occurs when the patient expresses a preference for outpatient care against the wishes of the remaining triad. The professionals permit this preference to supersede their judgment if they determine, based on their assessment and the outcomes of the SPI, that the patient poses no immediate danger in terms of suicidal potential or danger to others. This determination on suicidal potential relies on clinical experience rather than research evidence, as no superior alternative currently exists [28]. The healthcare professionals confirmed the final decision. Similarly, the professionals allow for the preference of the significant others for inpatient care against the wishes of the rest of the triad in certain cases. This decision is made by the professionals when there is a presence of suicidal risk or danger to others, or when there are problems with motivation. As with other determinations, the assessment of suicidal risk is guided by clinical experience rather than research evidence. An indication for outpatient care may be overridden if there are valid criteria for involuntary care. The healthcare professionals confirmed the final decision.

Previous research has highlighted that the integration of SDM practices is still in progress, resulting in a limited occurrence of wishes and needs of significant others [29]. This observation aligns with the findings of this study, where the professionals are reticent in allowing the preferences of either the patient or the significant others, indicating a possible gap in meeting the desires of involved parties within the SDM process. Triadic SDM in emergency psychiatry is possible [16], a conclusion that is confirmed by the results of this study. However, this domain remains relatively underexplored [30] and the development of strategies are needed to address conflicts between the parts of the triad.

A cross-sectional study examining the role of caregivers in psychiatric inpatient treatment [31] reported a low implementation of caregiver involvement, aligning with the findings of the present study. Earlier studies indicate that the degree of involvement of significant others is more difficult to implement than is commonly thought, and relies on the individual choices made by healthcare professionals [32, 33]. This understanding suggests that the frequency of significant others’ presence during assessments could potentially increase if healthcare professionals prioritize on involving them in the assessments.

### Limitations

Our results must be interpreted with caution since our analyses relied on retrospective and routinely collected data. Consequently, specific data regarding the preferences of the individual components within the triad were unavailable, highlighting the need for future research to delve into these aspects in greater detail.

In this study, we initially aimed to focus on the preferences of significant others rather than those of the patient. While the patient’s preferences should hold primary importance in triadic shared decision-making, our intention was not to prioritize the preferences of significant others. Instead, we sought to empirically investigate their relative importance within the decision-making process.

The assessments were conducted by multiple professionals, a characteristic inherent in the structure of emergency psychiatric service, where various professionals perform their duties. This diversity in professionals involved could potentially result in varying outcomes for the same cases, posing a potential source of bias that might have influenced the results obtained.

The SPI was utilized in this study to assess the severity of psychiatric illness. However, the original data distribution was skewed, potentially limiting the interpretability and validity of parametric statistical analyses. Dichotomization was applied to better handle skewed data and enable more robust analyses. While this approach does not inherently undermine the validity of the SPI as a measure of clinical outcomes, it may reduce the sensitivity to detect smaller yet meaningful variations in outcomes [34].

Schuster and colleagues [31] recommended a focus on interventions that prioritize involving caregivers in consultations. They proposed that a more comprehensive conceptualization of triadic SDM in mental health should be considered in a second step. The present study, aligning with these recommendations, also identifies a relatively low degree of involvement of significant others, thereby supporting this assertion.

This study found that triadic SDM occurred in half of the assessments, highlighting the need to promote triadic SDM in all assessments. The findings also demonstrate that triadic SDM is feasible in emergency psychiatric service assessments, even in the presence of discrepancies among the triad members. Discrepancies were found to occur less frequently than expected based on practical experience and can often be resolved by ensuring that all members of the triad are given the opportunity to express their perspectives. Future research should focus on identifying the characteristics of patients who disagree with the rest of the triad to develop more effective strategies for managing conflicts.

## Conclusions

In our exploration of the triadic SDM process, we observed that consensus was achievable in almost half of the assessments. Disagreements commonly arose when the patient sought outpatient care while the rest of the triad preferred inpatient care, or when significant others advocated for inpatient care while the remaining triad favoured outpatient care. The professionals’ recommendation held the most influence in determining the outcome, yet this recommendation could be disregarded if there were valid criteria necessitating involuntary care. To effectively manage conflicts in the triad, strategies need to be devised to address conflicts among the parts of the triad.

## Data Availability

The datasets used and/or analysed during the current study are available from the corresponding author on reasonable request.

## Abbreviations

SDM: Shared decision making
CRHT: Crisis Resolution and Home Treatment Team
EHR: Electronic health record
WGBO: The Netherlands Agreement on Medical Treatment Act
WMO: The Netherlands Medical Research Involving Human Subjects Act
WebRAAP: Web-based Registration and Advisory system for Acute Psychiatry
SPI: Severity of Psychiatric Illness
SPSS: Statistical Package for the Social Sciences
SMI: Serious mental illness
